# Reducing Soil-Emitted Nitrous Acid as a Feasible Strategy for Tackling Ozone Pollution

**DOI:** 10.1021/acs.est.4c01070

**Published:** 2024-05-16

**Authors:** Chaoyang Xue, Can Ye, Keding Lu, Pengfei Liu, Chenglong Zhang, Hang Su, Fengxia Bao, Yafang Cheng, Wenjie Wang, Yuhan Liu, Valéry Catoire, Zhuobiao Ma, Xiaoxi Zhao, Yifei Song, Xuefei Ma, Max R. McGillen, Abdelwahid Mellouki, Yujing Mu, Yuanhang Zhang

**Affiliations:** † Research Centre for Eco-Environmental Sciences, Chinese Academy of Sciences, Beijing 100085, China; ‡ State Key Joint Laboratory of Environment Simulation and Pollution Control, College of Environmental Sciences and Engineering, Peking University, Beijing 100871, China; § Max Planck Institute for Chemistry, Mainz 55128, Germany; ∥ Laboratoire de Physique et Chimie de l’Environnement et de l’Espace (LPC2E), CNRS—Université Orléans−CNES, Cedex 2 Orléans 45071, France; ⊥ Institut de Combustion Aérothermique, Réactivité et Environnement, Centre National de la Recherche Scientifique (ICARE-CNRS), Cedex 2 Orléans 45071, France

**Keywords:** O_3_ pollution, soil HONO emissions, nitrogen fertilizer, nitrification inhibitors

## Abstract

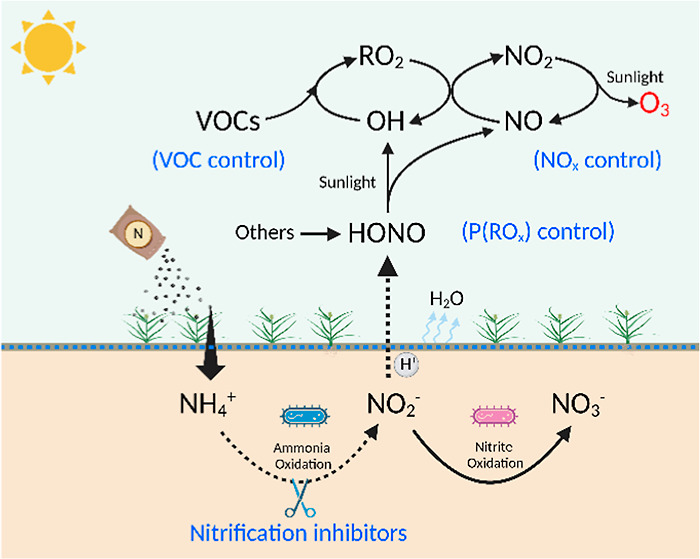

Severe ozone (O_3_) pollution has been a major air quality issue and affects environmental sustainability in China. Conventional mitigation strategies focusing on reducing volatile organic compounds and nitrogen oxides (NO_*x*_) remain complex and challenging. Here, through field flux measurements and laboratory simulations, we observe substantial nitrous acid (HONO) emissions (*F*_HONO_) enhanced by nitrogen fertilizer application at an agricultural site. The observed *F*_HONO_ significantly improves model performance in predicting atmospheric HONO and leads to regional O_3_ increases by 37%. We also demonstrate the significant potential of nitrification inhibitors in reducing emissions of reactive nitrogen, including HONO and NO_*x*_, by as much as 90%, as well as greenhouse gases like nitrous oxide by up to 60%. Our findings introduce a feasible concept for mitigating O_3_ pollution: reducing soil HONO emissions. Hence, this study has important implications for policy decisions related to the control of O_3_ pollution and climate change.

## Abstract 2

This study reveals significant HONO emissions from agricultural soils, impacting regional O_3_ pollution. Nitrification inhibitors show promise in reducing HONO emissions, offering potential air quality improvements.

## Introduction

Surface ozone (O_3_), a harmful pollutant, is associated with many adverse impacts on public health and plant growth, affecting the development of environmental sustainability.^1,2^ O_3_ is produced through chain photochemical reactions involving two major classes of precursors: volatile organic compounds (VOCs) and nitrogen oxides (NO_*x*_ = NO + NO_2_).^3,4^ Its production responds nonlinearly to its precursors, making it challenging to propose effective mitigation strategies. In efforts to mitigate O_3_ pollution, two chemical regimes are commonly recognized, namely, “NO_*x*_-limited” and “VOC-limited”. The NO_*x*_-limited regime refers to conditions where reducing NO_*x*_ would be most effective in reducing O_3_ production, while the VOC-limited regime describes situations where VOC reductions would be more beneficial. However, there are still large uncertainties in diagnosing the O_3_ formation regimes by models due to the incompletion of chemical mechanisms and uncertainties in the input data, such as emission information and meteorological predictions,^5^ constituting challenges in policymaking. Furthermore, achieving effective O_3_ mitigation requires a precise reduction ratio between VOCs and NO_*x*_. However, VOCs and NO_*x*_ are typically coemitted, leading to challenges in reducing NO_*x*_ and VOCs at a specific ratio. Otherwise, the reduction of both at an improper ratio may lead to an O_3_ increase. For instance, the COVID-19 lockdowns lead to significant simultaneous reductions in NO_*x*_ and VOCs while O_3_ shows clear enhancements on a national scale, suggesting the complexity and difficulties of mitigating O_3_ pollution by conventional strategies through reducing NO_*x*_ or VOCs.^6−8^

The chain reaction with O_3_ production is initiated and accelerated by primary radical production [*P*(RO_*x*_), including O_3_ photolysis and nitrous acid (HONO) photolysis] and propagated by the following radical cycling.^3,4,9^ Recent studies have highlighted the importance of *P*(RO_*x*_) in exacerbating O_3_ pollution.^10−12^ In particular, Wang et al.^11^ reported that O_3_ formation in Eastern China is sensitive to *P*(RO_*x*_), while Liu et al.^12^ demonstrated the significant contribution of primary radical sources, particularly HONO, to daytime O_3_ production in a high-O_3_ city in the North China Plain (NCP). These two studies highlight the need to recognize primary radical sources and indicate the potential role of *P*(RO_*x*_) reduction in mitigating O_3_ pollution in addition to conventional strategies.

The NCP is a region with severe O_3_ pollution,^13,14^ high radical levels, and high *P*(RO_*x*_).^15−19^ Among the primary radical sources, HONO plays a considerable or even dominant role, with a contribution of up to 90%.^17,20,21^ Our previous studies have indicated that agricultural fields in the NCP could be an important HONO source, especially after nitrogen fertilizer use (NFU).^22,23^ However, there are still no systematic studies to quantify NFU-induced HONO emissions, leading to uncertainties in assessing its impact on daytime radical and regional O_3_ production.^20,21^ Moreover, the lack of field flux measurements limits the advanced understanding of corresponding mechanisms of soil HONO emissions.^24^ Therefore, it is of important significance to conduct field flux measurements. Furthermore, reducing soil HONO emissions means less *P*(RO_*x*_), which could be an effective strategy for mitigating O_3_ pollution. However, to the best of our knowledge, no studies have been conducted to explore the control measure for reducing soil HONO emissions.

In this study, we conduct systematic field flux measurements, with coverage of several entire NFU-induced emission periods, and confirm the substantial HONO emissions induced by NFU in the NCP. We also quantify the impacts of soil HONO emissions on atmospheric oxidizing capacity and O_3_ pollution using a box model with constraints by comprehensive field measurements. Besides, we propose a new mechanism for soil HONO emissions through the combination of field flux measurements and laboratory simulations. Furthermore, we explore the potential control measures to reduce HONO emissions and hence mitigate O_3_ pollution, and estimate the impact of NFU-induced HONO emissions as well as their impacts on a global scale.

## Materials and Methods

### Field Measurements

Field flux measurements were conducted at the Station of Rural Environment, Chinese Academy of Science (SRE-CAS), which is surrounded by agricultural fields (38°71′N, 115°15′E) in Wangdu County, Hebei Province of China. Winter wheat and summer maize have been cultivated in the field for decades. The soil is classified as aquic Inceptisol, with a texture of sandy loam.^25^ Soil organic C and total N are 8.34–9.43 and 1.02–1.09 g kg^–1^, respectively. As a typical representative of agricultural regions, numerous comprehensive field campaigns, including measurements of greenhouse gas emissions and atmospheric compositions, have been conducted at this station.^20,21,25−27^ According to the cultivation habits of the local farmers, synthetic fertilizer (e.g., N(NH_4_Cl)/P_2_O_5_/K_2_O = 22%:8%:15%) is popularly used for summer maize planting. The fertilizer application rate in the NCP is from 120 to 729 kg N ha^–1^, and about 200–330 kg N ha^–1^ is typically used for the fields of nearby villages around the SRE-CAS station. Even higher fertilizer application rates (e.g., 3000 kg N ha^–1^ y^–1^) are frequently used for vegetable cultivation in the NCP.^28^

Soil HONO flux was measured by a twin open-top dynamic chamber (OTC) system, which has been detailed in the Supporting Information. The main field flux measurement campaign was conducted during 19 August–6 September 2016 with a typical fertilizer application rate of 247 kg N ha^–1^ (suggested by local farmers). Several other campaigns were conducted to reconfirm the NFU-induced soil HONO emissions and to explore the variations of soil HONO emissions with fertilizer application rates (Table S2). Other supporting measurements are described in Section S1 in the Supporting Information.

### Laboratory Experiments

A quartz incubator (inner diameter: 3 cm, length: 50 cm) with a jacket for circulating water (Figure S1) was used for laboratory experiments. A glass tank (length: 40 cm, width: 2 cm, height: 1 cm) that could be put inside the incubator was used to bear the soil samples (depth: 1 cm). At the outlet of the flow tube, HONO and NO were detected by LOPAP^30^ (or sometimes a stripping coil ion chromatography system^27^) and a NO analyzer (Thermo model 42i NO–NO_2_–NO_*x*_ analyzer, USA), respectively. The two HONO instruments showed good agreement in laboratory and field conditions, as reported in our previous study.^27^ Synthetic air (N_2_/O_2_ = 4:1) at a flow rate of 3.25 L min^–1^ was used to flush the flow tube. Before reaching the flow tube, the carrier gas passes through a relative humidity controller (RHC, details in Section S2 in the Supporting Information) to adjust its relative humidity.

Thanks to this platform, we studied the influencing factors of soil HONO emissions, including soil temperature, bacteria, fertilizer type, relative humidity of the flushing gas, and nitrification inhibitor (see details for each experimental design in Section S2 in the Supporting Information). For each experiment, 75 g soil samples collected at the SRE-CAS site^29^ (Section S2 in the Supporting Information) were filled into a glass tank with a surface area of 0.08 m^2^, humidified to 90% WHC by the water solutions of various fertilizers, and then incubated at a growth chamber (temperature: 20 °C; relative humidity: 80%; dark condition) before laboratory flux experiments.

### Model Simulations

A 0-D box model RACM v2 (regional atmospheric chemistry mechanism v2) was adopted to explore the influence of HONO emission from the fertilized soil on atmospheric HONO levels as well as O_3_ formation rates, as detailed in the Supporting Information and in previous studies.^31^ To explore the regional impacts, such as the enhancements in AOC and O_3_, soil HONO emissions were implemented into the RACM v2 model. Two scenarios were designed: with and without implementing the averaged diurnal HONO flux in the model. Comparison between the two scenarios can deduce the impact of *F*_HONO_ on O_3_ production.

## Results and Discussion

### Field Measurements of Soil HONO Flux and Atmospheric Composition

**Figure 1:**
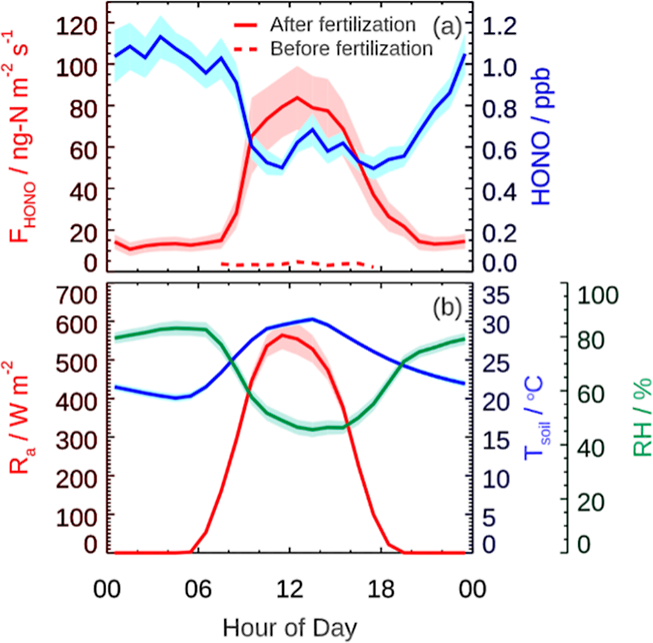
Diurnal profiles of soil HONO flux (*F*_HONO_), ambient HONO concentrations, solar radiation (Ra), soil temperature (T-soil), and atmospheric relative humidity (RH) measured in the summer of 2016. Error bars represent one-quarter of the standard deviation (±0.25σ).

Figure 1 displays diurnal profiles of soil HONO flux (*F*_HONO_) and associated parameters before and after fertilization. *F*_HONO_ remains below 3 ng N m^–2^ s^–1^ before fertilization. Similar levels of *F*_HONO_ were also observed at this site in 2021^32^ and other agricultural sites in China.^33,34^ Song et al.^32^ further observed a distinct diel profile of *F*_HONO_ before fertilization. In comparison, *F*_HONO_ increased significantly both during daytime and nighttime after fertilization and also exhibited regular peaks at noon. These daily peaks increased rapidly and reached a maximum of 348 ng N m^–2^ s^–1^ on the third day after fertilization (Figure S2), This level is 2 orders of magnitude higher than those measured from the same field before fertilization and >5 times greater than the reported values from other fields worldwide (<60 ng N m^–2^ s^–1^).^20^ Nevertheless, it is comparable to *F*_HONO_ from alkaline soils (up to 258 ng-N m^–2^ s^–1^) under laboratory studies, in which the emission is attributed to the nitrification process.^22,35^ The diurnal variation of *F*_HONO_ is similar to those of soil temperature (T-soil) and solar radiation (Ra) but opposite to ambient relative humidity (RH, Figure 1), indicating potential interactions between *F*_HONO_ and those parameters. It is worth noting that strong *F*_HONO_ is commonly observed after every NFU event, which can be obtained from our field flux measurements over multiple years (Table S2).

Before fertilization, a typical U-shape diurnal variation of ambient HONO has been frequently observed at this site.^20,36^ However, after fertilization, high *F*_HONO_ may result in significant changes in both ambient HONO levels and variations. Indeed, high unexpected HONO peaks, with an average of 0.7 ppbv, were observed at noon (Figure 1), in concert with the *F*_HONO_ peaks (84 ng m^–2^ s^–1^). This finding implies that the fertilized fields are the most significant daytime HONO source that reshapes the HONO diurnal variation. Similarly, during the summer of 2017, HONO enhancements were again observed after fertilization (Figure S3), revealing the reproducibility of NFU impacts on ambient HONO abundances. Additionally, there were notable increases in ambient O_3_ and hydrogen peroxide (H_2_O_2_) after fertilization (Figure S3), indicating the amplified role of enhanced HONO levels in atmospheric oxidizing capacity and O_3_ pollution.

### Insights on the Mechanism Based on Field Measurements

As illustrated in Figure S2, high *F*_HONO_ values were always observed during the daytime under a high or moderate soil water content (SWC). Previous laboratory experiments reported that the denitrification process could result in high soil HONO emissions at high SWC.^37,38^ During our field measurements, soil nitrate was increasing rapidly (Figure S4) after fertilization, suggesting an active nitrification process. This finding aligns with previous studies, in which soil NO and N_2_O emissions were attributed to the nitrification process in the NCP.^39^

Previous laboratory studies found that high HONO emissions occurred in the low soil water content range (10–40% WHC).^22,40^ However, our field measurements found that significant HONO emissions were mainly observed at a high SWC of ∼80% WHC (Figure S2). It is crucial to note that the measured soil water content represents the average moisture level of the surface soil down to a depth of 5 cm. However, the water content of the very surface layer, such as the top 1 mm, may be significantly lower. Moreover, elevated soil temperatures reduce the solubility of HONO and accelerate water evaporation.^3^ Additionally, low RH at noon can further hasten water evaporation. As a result, evaporation from this surface layer can markedly alter soil surface properties,^37,41−43^ including microscale pH^44^ and equilibrium HONO concentration,^23,43^ leading to an increase in HONO emissions. Therefore, the combined effect of rising temperatures, coupled with decreasing air RH, stimulates HONO emissions through the interaction of reduced HONO solubility and accelerated water evaporation, which could explain the observed diurnal variations of *F*_HONO_. Together with the below laboratory results, an advanced mechanism of soil HONO emissions is proposed.

### Key Factors Driving Soil HONO Emissions

**Figure 2:**
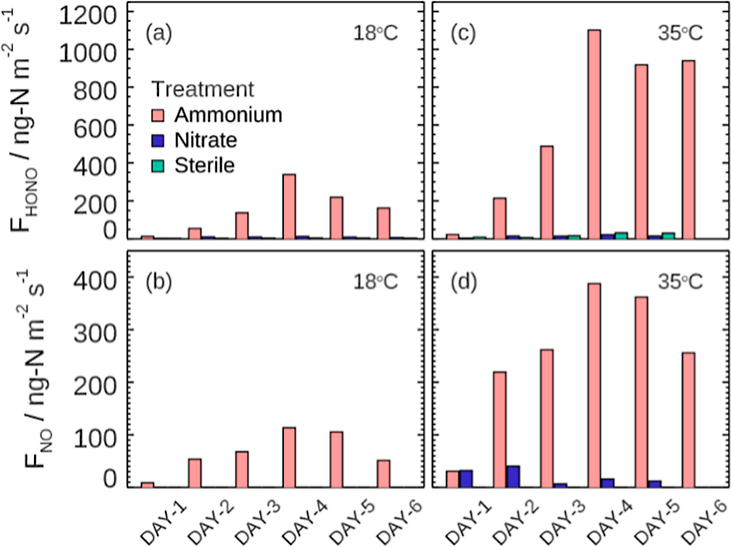
Emissions of HONO (*F*_HONO_) and NO (*F*_NO_) at 18 [panels (a,b)] and 35 °C [panels (c,d)]. Soil samples were in parallel treated by sterilization + NH_4_Cl (sterile), KNO_3_ (nitrate), and NH_4_Cl (ammonium).

To explore the key factors driving soil HONO emissions, we conduct a series of incubator experiments by incubating the agricultural soil samples. Simultaneous measurements of NO emissions (*F*_NO_) are also conducted, as they are known to generally coexist with *F*_HONO_.^22,40^Figure 2 exhibits *F*_HONO_ and *F*_NO_ under different treatments. *F*_HONO_ and *F*_NO_ from NH_4_Cl-treated soil samples substantially increase and reach their maximums on the fourth day after fertilization, similar to field measurements which show peak emissions on the third day after fertilization (Figure S2). In contrast, much smaller *F*_HONO_ and *F*_NO_ are observed for the sterile + NH_4_Cl- and KNO_3_-treated soil samples, which is consistent with the observed *F*_HONO_ that does not increase with soil nitrate concentration in the field measurements (Figures S2 and S4). Consequently, an inference that HONO emissions are primarily derived from ammonium fertilizer as opposed to nitrate can be drawn. This inference can be also supported by the results of parallel NH_4_Cl treatment experiments with and without the addition of nitrification inhibitors to block the ammonia oxidation (via NH_4_^+^ → NO_2_^–^) process, e.g., more than 90% reduction of HONO and NO emissions from the ammonium treatment with the presence of DCD (dicyandiamide, a nitrification inhibitor).

Temperature dependence is also explored. Both *F*_HONO_ and *F*_NO_ increase by a factor of ∼3 at the soil temperature of 35 °C as against 18 °C (Figure 2), resulting from the accelerated nitrification process^22^ and surface water evaporation (see the Results and Discussion section). It is worth noting that the temperature dependence experiments also suggest the impact of SWC changes. As shown in Figure S6, each time the experimental temperature is switched from 18 to 35 °C, *F*_HONO_ rapidly increases. However, *F*_HONO_ does not return to a similar level when the temperature is switched back, indicating the additional impact of SWC changes in soil HONO emissions.

Figure S7a illustrates the impact of air humidity on soil HONO emissions. When the fertilized soil sample is flushed by humidified air, *F*_HONO_ stabilizes in 30 min. Surprisingly, when switching the flushing gas to dry air, *F*_HONO_ rapidly shows a pulse peak, followed by a fallback and then a slight increase during the drying process. On average, *F*_HONO_ increases by a factor of 3 during the dry air flushing period compared to the humidified air flushing period, indicating the significant effect of the surface drying process on soil HONO emissions. This result highlights the importance of water exchange between the soil surface and atmosphere in regulating HONO emissions. We, therefore, conduct quantitative investigations of the relationship between the surface drying process and HONO emissions under different RH conditions. The time series of *F*_HONO_ during this experiment is shown in Figure S8. In the RH range of 60–100%, *F*_HONO_ increases as RH decreases and can reach a generally stable level for each RH gradient. However, if RH continues to reduce, *F*_HONO_ still increases but cannot reach a stable level. This is due to relatively larger SWC changes under lower RH conditions. Despite that, very high correlations (*R*^2^ = 0.98, Figure S7b) are still found between *F*_HONO_ and soil water loss rate (*E*_water_), indicating the remarkable impact of surface water exchange on soil HONO emissions.

At high temperatures, HONO solubility is lower according to Henry’s law, the nitrification process is more active^22,37^ to produce NO_2_^–^, and surface water evaporation is more rapid than at low temperatures. Hence, higher emissions are expected at higher temperatures, which could explain the significant increase in emissions when increasing soil temperature from 18 to 35 °C (Figure 2). The syngeneic effect of ambient RH and T-soil governs the diurnal variations of HONO solubility, nitrification activities, and surface drying process, which collectively explain the observed diurnal variations of *F*_HONO_ (Figures 1 and S2).

**Figure 3:**
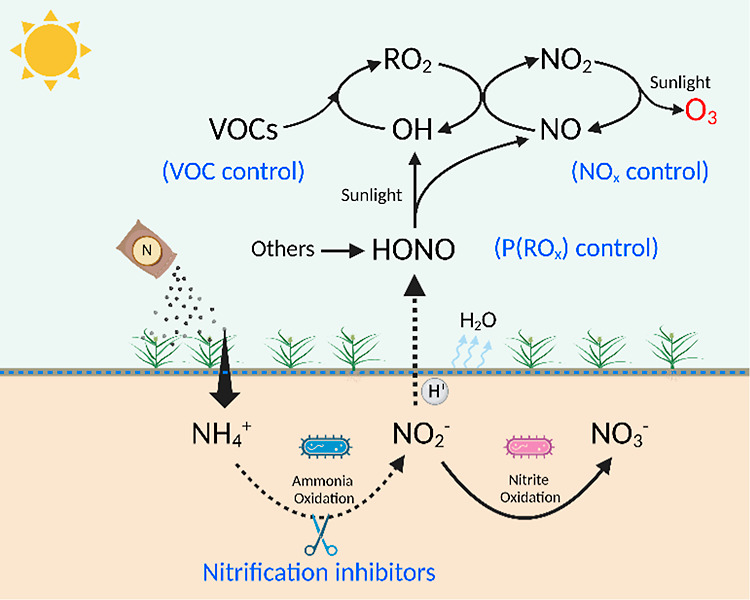
Schematic plot of soil HONO emission mechanism, impacts on O_3_ pollution, and control measures. Conventional O_3_ mitigation mainly focuses on VOC control or NO_*x*_ control and here, we propose a feasible concept of controlling primary radical sources [*P*(RO_*x*_), e.g., HONO].

Therefore, our results provide valuable insights into the complex process driving HONO emissions from soil surfaces (Figure 3). On the one hand, the application of nitrogen fertilizers stimulates the microbial process such as the ammonium oxidation process with the production of nitrite. This accumulation of soil nitrite serves as a crucial precursor for HONO emissions. It is important to note that microbial processes can be influenced by various factors, including soil pH, temperature, soil water content, etc. Understanding these factors is essential for accurately predicting HONO emissions under different environmental conditions. Additionally, genetic analysis will benefit the understanding of the role of various microbial processes in soil HONO emissions. On the other hand, soil temperature and ambient RH play key roles in modulating the surface drying process, affecting soil surface properties at a microscale. Higher temperatures and lower relative humidity levels promote faster soil surface drying, potentially leading to enhanced HONO emissions. This relationship underscores the importance of considering meteorological conditions when assessing HONO fluxes from soil surfaces.

Several laboratory studies, such as those conducted by Wang et al.^45^ and Song et al.,^46^ have collected different types of soil samples in different regions across China and observed significant HONO and NO_*x*_ emissions from those soil samples. Notably, Song et al. also found that ammonium fertilizer could largely increase emissions through enhancing the nitrification process. Field flux measurements are needed to quantify these emissions on a national scale. We particularly emphasize the link between water exchange at the soil–air interface and the release of soil nitrification-originated nitrite as HONO, as this may also apply to other water-soluble gas emissions, such as ammonia (NH_3_). The released HONO maintains a high daytime HONO level, which acts as a strong OH source to accelerate daytime photochemistry, resulting in the formation of secondary pollutants, such as O_3_ pollution.

### Impact on O_3_ Pollution

Figure S9 demonstrates the impact of *F*_HONO_ on the HONO budget. The default mechanism, which only considers NO + OH as the HONO source, predicts a HONO concentration of only 0.07 ppbv, more than 1 order of magnitude lower than the observations (1.21 ppbv). The inclusion of *F*_HONO_ significantly improves the model’s performance, as the predicted HONO level of 1.28 ppbv and variation are very similar to observations, suggesting the dominant role of *F*_HONO_ in the HONO budget. This is in agreement with our ambient HONO measurements, i.e., unexpectedly noontime HONO peaks (0.7–1.7 ppbv) were observed at this site during the summers of 2016 and 2017 after fertilization (Figures 1 and S3).

**Figure 4:**
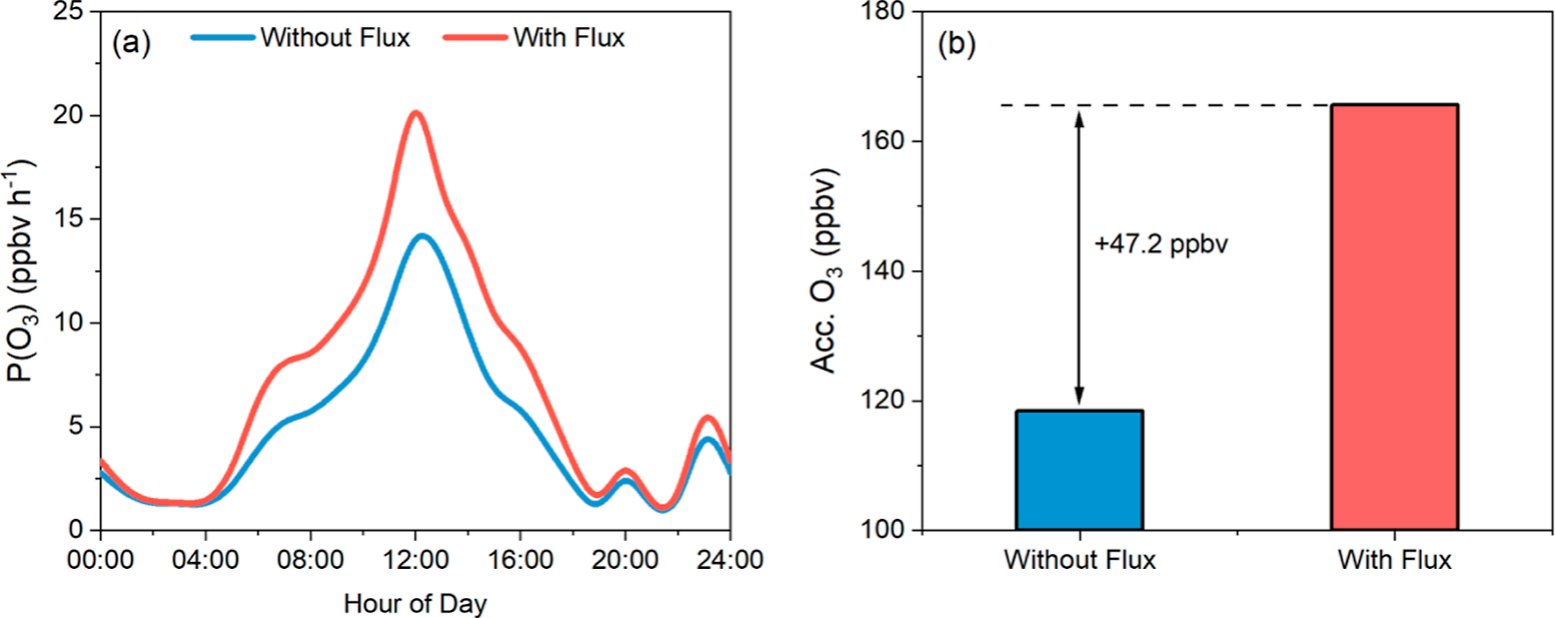
Impact of soil HONO emissions on the O_3_ production rate [*P*(O_3_), panel (a)] and average daily accumulated O_3_ production [Acc. O_3_, panel (b)].

The high level of ambient HONO maintained by *F*_HONO_ leads to increased OH production and a stronger atmospheric oxidizing capacity, resulting in the formation of secondary pollutants such as O_3_. Figure 4a demonstrates that the inclusion of *F*_HONO_ leads to a substantial increase in the O_3_ production rate (*P*(O_3_)), which can reach up to 8.5 ppbv h^–1^ at noon. Additionally, the average daily accumulated O_3_ production increases by 37% (47.2 ppbv), highlighting the significant impact of *F*_HONO_ on O_3_ production. Furthermore, significant O_3_ enhancements caused by NFU were observed at this agricultural site (Figure S3), as well as other sites in the NCP.^20^ This emphasizes the substantial impact of *F*_HONO_ on regional O_3_ pollution, which has been largely overlooked.

## Atmospheric Implications

### Reactive Nitrogen Budget and Greenhouse Gas Emissions

This study provides systematic continuous flux measurements after NFU events, enabling the estimation of nitrogen loss via HONO emissions (EF(HONO)). Based on our measurements, about 0.21% of applied nitrogen is lost via HONO emissions within 17 days after fertilization. We note that the EF(HONO) of 0.21% represents a minimum due to the limitations of the measurement period. Further flux measurements covering the entire growing season are needed to determine a precise EF(HONO). The obtained EF(HONO) is at a similar magnitude to other nitrogen gases (e.g., NO and N_2_O: ∼1.0%),^47−51^ and hence, the estimation of global NFU-induced HONO emission is crucial, as its photolysis can produce both OH and NO_*x*_, perturbing the atmospheric self-cleaning capacity and affecting regional air pollution.

Currently, NFU is commonly conducted for agricultural activities worldwide to increase crop yields and has shown an increasing trend since the invention of ammonia synthesis in the 1910s,^52^ constituting an important reactive nitrogen source on a global scale. In the NCP, NFU events occur regularly (>4 times per year for agricultural fields) with a higher application rate of 290 kg N ha^–1^ (data source: China Statistical Yearbook 2019) compared to a world average of 75 kg N ha^–1^. In vegetable-planting areas near megacities, even much higher fertilizer application rates (e.g., ∼3000 kg N ha^–1^) are used with a higher application frequency.^28^ The high application rate and the large NFU in China (24 Tg, around one-quarter of world fertilizer consumption of 108 Tg, data source: Statista) suggest considerable NFU-induced impacts on atmospheric composition. Assuming a lower limit of EF(HONO) of 0.21% for all types of N fertilizers, NFU-induced HONO emissions are estimated to be 0.05 and 0.23 Tg N for China and the globe, respectively. By far, emission inventories only potentially consider soil NO_*x*_ emissions but not HONO emissions. Such amounts of NFU-induced HONO emissions correspond to 6.5 and 10% of agricultural NO_*x*_ emissions in China (0.77 Tg N^53^) and the globe (2.26 Tg N^54^), indicating the overlooked role of soil HONO emissions in exacerbating regional air quality and the urgency of exploring corresponding emission control measures. One should also bear in mind that here the rough estimation of total NFU-induced HONO emissions needs to be further updated with more field constraints on the EFs. Many influencing factors on the EF, such as soil types, climatic conditions, fertilizer type, and application rate are still poorly understood. Further studies are still needed to address the uncertainties in EFs, the influencing factors, and the reactive nitrogen budget.

### Emission Reduction Measures

As demonstrated above, nitrification is the major source of soil nitrite, the precursor of HONO. Applying nitrate-based fertilizers may indeed reduce reactive nitrogen emissions, as suggested by our laboratory results. However, it is important to note that nitrate-based fertilizers have been reported to cause other problems such as groundwater pollution and safety concerns. Nitrification inhibitors, such as DCD (C_2_H_4_N_4_) can suppress nitrification activity by blocking the formation of hydroxylamine (NH_2_OH), the precursor for soil NO_2_^–^. It has been suggested to reduce N_2_O emissions^55^ and HONO and NO emissions as well.

Figure S10 shows the results of DCD impacts on HONO emissions. HONO concentrations in the incubator rapidly increase with incubation days after fertilization and reach their peak of about 100 ppbv on the third day after fertilization. Similar NO variations are observed, but at a lower level (peak on the second day after fertilization; maximum concentration: 60 ppbv). In contrast, fertilized soil samples with additional treatment of 5 or 10% DCD (relative to applied nitrogen) show considerable HONO and NO emissions only on the second and third days after fertilization. Maximums of HONO and NO emissions are >6 times lower with an additional 10% DCD treatment. On average, with 5% DCD accompanied by nitrogen fertilizer application, the reduction efficiencies in HONO and NO emissions are 78 and 70%, respectively. The reduction efficiencies increase to 90 and 86% for HONO and NO, respectively, for 10% DCD treatments. Additionally, our previous study observed DCD-induced N_2_O reduction by 66% in the NCP region.^47^ Moreover, nitrification inhibitors play a role in alleviating soil acidification by reducing nitrification processes, which are significant drivers of soil acidification in Chinese croplands.^56^ Furthermore, a reduced nitrification process improves the nitrogen use efficiency, resulting in benefits for the crop yields.^57^ Thus, the control strategies proposed in this study can reduce soil HONO and N_2_O emissions synergistically, which would be beneficial for environmentally sustainable development and lead to cobenefits of air quality, public health, soil health, crop yields, and global climate. However, the use of nitrification inhibitors poses the risk of increasing ammonia emissions from agricultural soil, as they maintain high ammonium concentrations in the soil, which could lead to increased ammonia volatilization.^58,59^ We note that further worldwide assessments are needed to fully comprehend the impacts of nitrification inhibitors on soil–atmosphere exchanges, soil properties, and global implications for air quality and climate.

Taken together, this study proposes a feasible concept of reducing primary radical sources (e.g., soil-emitted HONO) to mitigate O_3_ pollution (Figure 3). We also demonstrate the great potential of nitrification inhibitors in reducing emissions of reactive nitrogen (HONO and NO_*x*_) and greenhouse gases (N_2_O) and thus mitigating both regional air pollution and global climate.

## Data Availability

All data for the figures in the main tests and Supporting Information are available from the lead contact upon reasonable request.

## Supporting Information Available

Detailed information about field measurements; laboratory experiments; box model; HONO emission factor; summary of instruments used in the field campaigns; maximum *F*_HONO_ and fertilizer application rates; diagram of the flow tube; time series of the measured HONO flux and related parameters; average diurnal profiles of HONO, O_3_, and H_2_O_2_; relative contributions of HONO and O_3_ to primary OH production and the measured HONO flux (2017) measured at the SRE-CAS site; measured soil pH and NO_3_^–^ concentration during the campaign; correlation between HONO flux during gradient RH experiments; results of the temperature dependence experiment; influence of water evaporation on HONO emissions from NH4Cl-fertilized soil sample; time series of *F*_HONO_ measured during gradient RH experiments; results of model simulations with/without HONO flux in comparison with observations; and impacts of a nitrification inhibitor on soil HONO and NO emissions (PDF)
