# Supplementary material for: Reducing Soil-Emitted Nitrous Acid as a Feasible Strategy for Tackling Ozone Pollution
Source: Environ Sci Technol. 2024 May 16;58(21):9227–35. doi: 10.1021/acs.est.4c01070 (PMC11137860; doi:10.1021/acs.est.4c01070)
Supplement: Supplementary file 1 — es4c01070_si_001.pdf [file es4c01070_si_001.pdf]

Supplemental information for

Reducing Soil Emitted Nitrous Acid as a Feasible Strategy for Tackling Ozone Pollution

Chaoyang Xue[1, 3, 4, #, *], Can Ye[2, #], Keding Lu[2, *], Pengfei Liu[1], Chenglong Zhang[1], Hang Su[3], Fengxia Bao[3], Yafang Cheng[3], Wenjie Wang[3], Yuhan Liu[2], Valéry Catoire[2], Zhuobiao Ma[1], Xiaoxi Zhao[1], Yifei Song[1], Xuefei Ma[2], Max R. McGillen[5], Abdelwahid Mellouki[5], Yujing Mu[1, *], Yuanhang Zhang[2]

[1] Research Centre for Eco-Environmental Sciences, Chinese Academy of Sciences, Beijing 100085, China

[2] State Key Joint Laboratory of Environment Simulation and Pollution Control, College of Environmental Sciences and Engineering, Peking University, Beijing, 100871, China

[3] Max Planck Institute for Chemistry, Mainz 55128, Germany

[4] Laboratoire de Physique et Chimie de l'Environnement et de l'Espace (LPC2E), CNRS–Université Orléans–CNES, Cedex 2, Orléans 45071, France

[5] Institut de Combustion Aérothermique, Réactivité et Environnement, Centre National de la Recherche Scientifique (ICARE-CNRS), Cedex 2, Orléans 45071, France

*Correspondence:

Chaoyang Xue, ch.xue@mpic.de

Keding Lu, k.lu@pku.edu.cn

Yujing Mu, yjmu@rcees.ac.cn

Number of Pages: 15

Number of Texts: 4

Number of Tables: 2

Number of Figures: 10

1. Field Measurements

Flux Measurements. A twin open-top dynamic chamber (OTC) system was used to measure soil HONO flux. A detailed design can be found in our recent studies[1,2]. Briefly, two Teflon chambers, one experimental chamber whose base covers the soil and one reference chamber whose base was sealed by Teflon film, were simultaneously flushed with ambient air at a constant flow of 20 L min^{-1}. Both chambers were open-top so that the condensation of water, which can cause significant HONO loss, was avoided. Hence, soil HONO flux could be inferred from the difference in HONO concentrations in the two chambers, as shown in below equation:

$$F_{HONO\text{-}N}=\frac{(C_{Exp}\text{-}C_{Ref})\times F_{flush}\times M_N\times P}{R\times T\times S}\times\frac{1}{60} \quad (1)$$

$F_{HONO\text{-}N}$: HONO emission flux based on N, ng m^{-2} s^{-1};

C_{Exp}: HONO concentration in the Exp-chamber, ppbv;

C_{Ref}: HONO concentration in the Ref-chamber, ppbv;

F_{flush}: flushing flow, L min^{-1};

M_N: molar mass of N, g mol^{-1};

P: atmospheric pressure, kPa;

R: ideal gas constant, L kPa mol^{-1} k^{-1};

T: thermodynamic temperature, K;

S: soil area covered by the chamber, m^{-2}.

The OTC system was first successfully combined with a wet chemical HONO measurement method with a time resolution of 1 h, the results of which are presented in this study[1,3]. Further applications on flux measurements of other reactive species (i.e., NH_3, NO) and greenhouse gases (e.g., N_2O, CH_4, CO_2) are foreseeable.

Measurements of Atmospheric Composition. During the field campaigns, the concentrations of atmospheric NO_x (NO, NO_2) and NO_y (the sum of the reactive nitrogen oxides, including NO, NO_2, PAN, HNO_3, HONO, particulate nitrate, etc.) were measured by the Model 42i-NO_x analyzer (Thermo Scientific, USA) and Model 42i-NO_y analyzer (Thermo Scientific, USA), respectively. H_2O_2 was measured by a commercial instrument (AL2021 AEROlaser, Germany). $PM_{2.5}$ was measured by a standard tapered element oscillating microbalance system (TEOM 1400A, Thermo Scientific, USA). See Table S1 for the list of instruments involved in our campaigns.

Measurements of Meteorological Parameters. The total solar irradiance, ambient temperature, relative humidity (RH), wind speed, wind direction, and soil temperature (depth: 5 cm) were automatically measured and recorded by an auto meteorological station at the SRE-CAS station.

Measurements of Soil Parameters. During the HONO flux measurement period, soil

parameters were also measured synchronously. Three soil samples were collected every two days to measure the concentrations of soil water content (% WHC), soil nitrite, soil nitrate, and soil pH. Each soil sample was divided into three parts: one for measuring the SWC by quantifying the soil weight before and after heating in an oven at 105 °C for 24 hours, one for measuring the soil pH based on the method of ISO 10390:2005-2012, and the other for measuring the soil nitrite and nitrate concentration based on the method of ISO/TS 14256-1:2003 (E).

For comparison with the values of soil water content (SWC) reported by previous studies, the SWC in this study was calculated based on the water-holding capacities (WHC), which is derived from the equation:

$$WHC= \frac{m_{sat\text{-}water}}{m_{dry\text{-}soil}} \quad (1)$$

where $m_{sat\text{-}water}$ is the weight of soil water under the condition of the soil saturated with water and $m_{dry\text{-}soil}$ is the mass of the soil after heating at 105 °C for 24 hours. The SWC in % WHC is the proportion of the weight of soil water to $m_{sat\text{-}water}$.

2. Laboratory Experiments

Soil Samples. At the end of the flux measurement campaign in 2016, the surface soil (depth: 0-5 cm) in the agricultural field around the SRE-CAS site was collected by a scoop into a knitted bag. Soil samples were then sieved by a 2-mm sieve and stored in a refrigerator at 4 °C. We measured the soil property every two days during the campaign. The last measurement of the soil property (pH: 8.0; NH_4^+; 1.37 mg kg^{-1}; NO_3^-: 146.91 mg kg^{-1}) can be treated as the initial soil property for laboratory experiments. This would not cause significant uncertainties because: a) soil pH is a stable parameter as observed during this campaign (Figure S4), and b) the initial nitrogen nutrient is much lower than those after fertilizer treatment in the laboratory experiments. Besides, the initial SWC of soil samples was measured by quantifying the soil weight before and after heating in the oven at 105 °C for 24 hours.

Relative Humidity Controller (RHC). The RHC system consists of a thermostatic water bath, a washing bottle with ultrapure water inside, two three-way valves, and two flow controllers. Briefly, the dry carrier gas was split into two channels. Each channel was regulated with a mass flow controller, one of which went through a glass gas wash bottle to become humidified and then mixed with the other channel before entering the flow tube. The thermostatic water bath could keep the temperature of the washing bottle stable (±0.5 ˚C) so that the carrier gas could get stable relative humidity (RH). Gradient RHs could be obtained by changing the ratio of the two embranchments. Laboratory tests found that the RH of the carrier gas and the soil temperature could reach the target values in 2 min (±3%) and 3 min (±1˚C), respectively.

Influence of Soil Temperature, Soil Bacteria, and Fertilizer Types. Well-mixed soil samples were divided into three parts (defined as S1, S2, and S3). S1 was sterilized by an autoclave at a given pressure (0.1 MPa) and temperature (121 °C) for 1 h. This could kill the microbes in the soil but didn't significantly change other properties of the soil. Then, S1, S2, and S3 were fertilized by NH_4Cl, NH_4Cl, and KNO_3 solutions, respectively, with a constant fertilizer application rate of 400 mg-N kg^{-1} soil. This fertilizer application rate is equal to ~250 kg-N ha^{-1} for field conditions. To keep the variation of soil water content similar to field conditions, i.e., small changes in the measured soil water contents within one week after fertilization (80-90%WHC, Figure S2), soil samples were initially wetted to 90%WHC and the water loss was replenished through spraying water on the soil surface after daily measurements.

During each incubation day, HONO and NO emissions were simultaneously measured for 1 h at 18 °C followed by 1 h at 35 °C. Soil temperature was controlled by circulating water from two thermostat water baths set at 18 and 35 °C, respectively. RH of the flushing gas was 61.7%. Results are shown in Figure 2 in the main text.

Regarding temperature, we designed another experiment. HONO emissions from a fertilized soil sample (incubation time: 48 h) were determined, with temperature switching between 18 and 35 ºC for three cycles. Results are shown in Figure S6.

Influence of RH and Surface Water Exchange. Three NH_4Cl-treated (400 mg-N kg^{-1}) soil samples were used to explore the impacts of the RH of the carrier gas. For each sample, HONO emission was measured for 1 h. The RH of the carrier gas was set at 48.8% for the first 30 min measurement. After that, it was set at 0% (dry synthesis air) for another 30 min. Results are shown in Figure S7.

To quantify the impact of soil water evaporation, HONO emission from NH_4Cl-treated soil samples was measured under gradient RH levels. No visible water film was formed on the inner surface of the flow tube. During each RH step, soil water loss (Δm) was obtained through the difference in the weight of the glass tank (containing the soil sample) before and after each experiment. Δm was larger than 1 g for each RH step, which can be easily detected by a balance with a precision of 0.01 g.

Weight loss caused by gas emissions other than water evaporation has negligible impacts on Δm as they are several orders of magnitude lower than water loss. The average water evaporation rate (E_{water} in g min^{-1}) could also be obtained according to Δm divided by experimental time. During those experiments, HONO emissions at each RH step were measured for 25 min at 35 °C. Results are shown in Figures S7 and S8.

Influence of Nitrification Inhibitors. Well-mixed soil samples were divided into three parts (defined as S4, S5, and S6). All the samples were treated with 100 mg-N (NH_4Cl) kg^{-1} soil and wetted to 90% WHC. S5 and S6 were additionally treated with 5 and 10 mg-DCD kg^{-1}, respectively. HONO and NO emissions from those three samples were measured every day for 30 min. The soil temperature was controlled at 25 °C, and dry air was used in the experiment to accelerate HONO release from soil samples, in order to assess the performance of DCD. Results are shown in Figure S11.

Chemicals. Chemicals used in this study, including NH_4Cl (>99.5%), KNO_3 (>99%), dicyandiamide (DCD, >99%), $NaNO_2$ standard solution (0.1 M), and N-(1-Naphthyl) ethylenediamine dihydrochloride (>98%) were purchased from Sigma-Aldrich. Other chemicals, including hydrochloric acid (36-38%), and KCl (>99.8%), were purchased from Sinopharm Chemical Reagent Co., Ltd.

3. Model Simulations

A 0-D box model RACM2 was adopted to explore the influence of HONO emission from the fertilized soil on atmospheric HONO levels as well as O_3 formation rates. The mechanism in the model contains 17 stable inorganic compounds, 4 inorganic intermediates, 55 stable organic compounds, and 43 organic intermediates, as detailed and documented in previous studies[4], which can be constrained by field measurements or emission information. By default, the mechanism contains only the gas phase reaction of NO+OH as a HONO source.

However, due to the limitation of available field data, model simulations could not be run for the summer of 2016. Instead, we ran box model simulations for the summer of 2014 when a comprehensive field campaign (including measurements of VOCs, radicals, O_3, NO_x, J-values, etc.) was conducted at a site near our station (about 2 km)[5]. Because similar fertilizer types and application rates were used in this region every summer for planting summer maize, similar HONO emission variations were also expected each year after planting summer maize. This could be also confirmed by our several field flux measurements in the summers of 2015-2017. The model was constrained by the field observation data in the summer of 2014 at this site, as detailed in Liu et al.[5]

To quantify the impact of F_{HONO} on O_3 production, two scenarios were designed: with and without implementing the averaged diurnal HONO flux in the model. Other HONO sources like heterogeneous reactions and other primary emissions are not considered as the comparison between the two scenarios we set can determine the impact of F_{HONO} on O_3 production. Similar to gases like CO, NO, and SO_2 that can be constrained by emission flux from emission inventories, the observed F_{HONO} can also be integrated into the model as a ground HONO source, which affects the HONO concentration as well as the follow-up chemistry.

We didn't simulate the O_3 concentrations because it is significantly affected by regional transport and convection, which are not considered in the box model. The box model can work well in simulating in-situ atmospheric chemistry like O_3 production rate but for the O_3 concentrations, we call for further model studies utilizing regional chemistry transport models.

4. HONO Emission Factors

As shown in Table S2, only two campaigns in summer 2016 (P1: 27 Jun.-24 Jul. 2016, and P2: 19 Aug.-6 Sept. 2016) are relatively longer and they can be preliminarily used for EF(HONO) calculations. The EFs in P1 and P2 are 0.64% and 0.21%, respectively. The higher EF in P1 than in P2 could be due to the influencing factors: a) higher fertilizer application rate in P1 (330 kg-N ha^{-1}) than in P2 (247 kg-N ha^{-1}) and the peak emissions (and possibly accumulated emissions) are exponentially correlated with fertilizer application rate (Table S2); b) during P2 summer maize has been planted for more than 1 month and could absorb more nutrient than in P1. In addition, the different fertilizer application methods (deep fertilization during machine sowing and spreading fertilizer on the soil surface) also affect the emissions. For instance, in our recent study, Song et al.[2] found that burying the fertilizer to a depth of ~10 cm below the ground surface could reduce peak HONO emissions by 31% compared to spreading fertilizer on the soil surface.

The EFs for the laboratory experiments with ammonium fertilizer treatment were also calculated. They are 0.2% and 0.8% when using the average flux at 18°C and 35°C, respectively. Although they are at a similar level to field measurements in this study or Song et al.[2], the difference in laboratory and field conditions still leads to uncertainties in laboratory-derived EFs. For instance, the thin soil depth (1 cm) used in laboratory experiments may favor the release of HONO from the soil samples to the incubator air. On a larger scale, such as national or global scales, soil types, and climatic conditions may also show impacts on EFs. Taken together, further studies are still needed to address the uncertainties in EFs and the influencing factors.

Tables

Table S1: Instruments used in the field campaigns.

Analyzer	Parameter	Detection limit	Measurement principle	Resolution
Thermo Scientific Model 42i-Y, USA	NO/NO_y	0.5 ppbv	Chemiluminescence	1 min
Thermo Scientific Model 42i, USA	NO/ NO_x	0.4 ppbv	Chemiluminescence	1 min
SC-IC	HONO	20 pptv	Ion chromatography	1 hour
LOPAP-03, QUMA, Germany	HONO	5 pptv	Long-path liquid absorption	1 min
Thermo Scientific Model 49i, USA	O_3	0.5 ppbv	UV absorption	1 min
Thermo Scientific 1405 TEOM, USA	$PM_{2.5}$	2 μg m^{-3}	Tapered element oscillating microbalance	1 min
AL2021 H_2O_2 analyzer, AEROlaser, Germany	H_2O_2	0.1 ppbv	liquid chemistry fluorescence	1 min
Thermo Scientific Model 43i, USA	SO_2	0.5 ppbv	Pulsed fluorescence	1 min
Seal Analytical AutoAnalyzer 3, USA	Soil NO_3^-	0.1 μg L^{-1}		
Mettler Toledo Delta 320 pH Analyzer, USA	soil pH	0.1		

Table S2: Maximum F_{HONO} and fertilizer application rates.

Measurement Period	Fertilization Rate (kg-N ha^{-1})	F_{HONO}_max (ng-N m^{-2} s^{-1})	F_{HONO}_average (ng-N m^{-2} s^{-1})
10-14 Aug. 2015	0	3.2	1.9±0.9[1]
10-14 Aug. 2015	45	40.4	21.0±11.3[1]
27 Jun.-24 Jul. 2016	330	1515	187±261[1]
11-12 Aug. 2016	0	4.6	3.5±0.7
19 Aug.-6 Sept. 2016	247*	348	34.5±53.2
13-21 Jun. 2017	180	161	19.9±26.9

*: the typical fertilizer application rate by local farmers

Figures

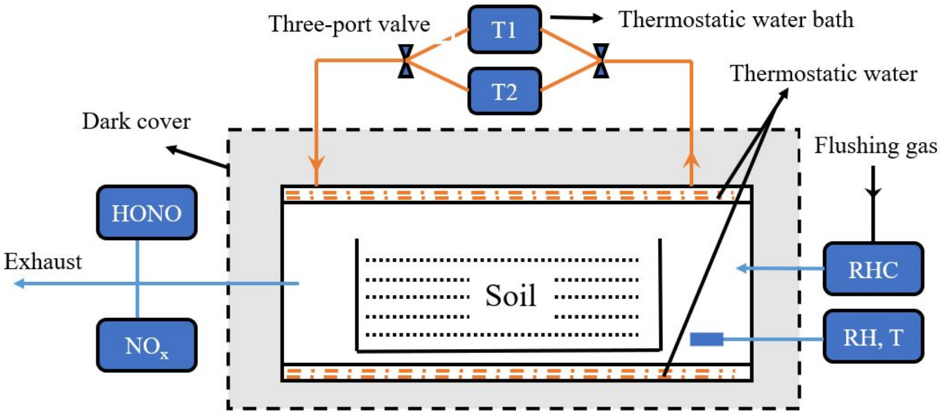

Figure S1: Diagram of the flow tube. T1 and T2 represent the two thermostatic water baths; RHC represents the relative humidity controller system.

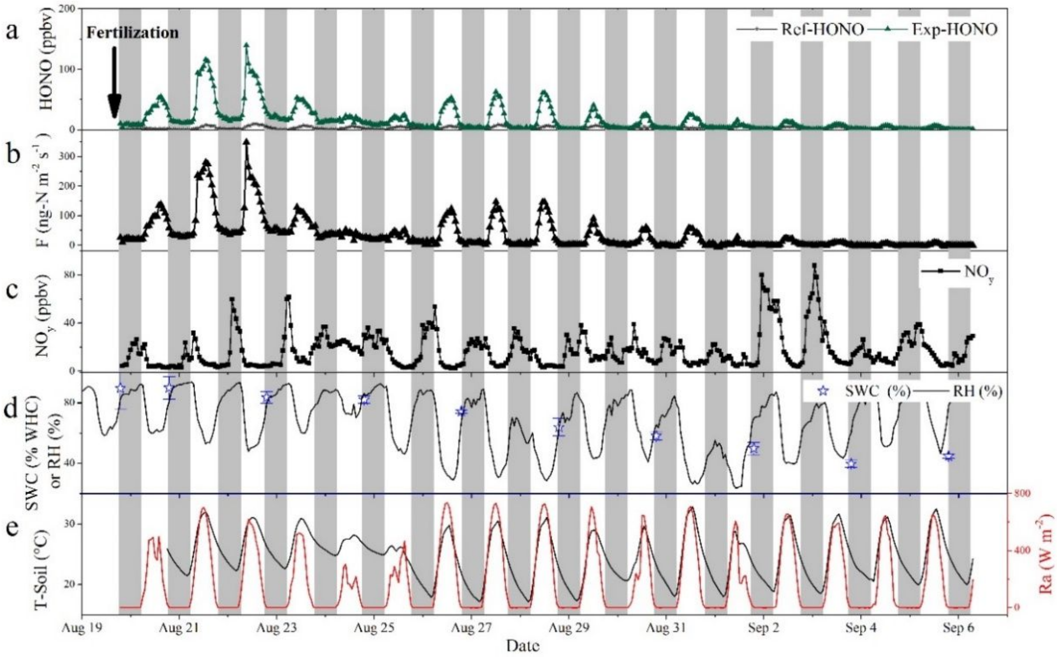

Figure S2: Time series of the measured HONO flux and related parameters in 2016. a: HONO concentrations in the experimental chamber (Exp-HONO) and reference chamber (Ref-HONO). Note that information about ambient HONO was presented in Figure S3; b: Soil HONO fluxes (F_{HONO}); c: ambient concentrations of total reactive nitrogen (NO_y); d: soil water content (SWC in %WHC) and atmospheric relative humidity (RH); e: soil temperature (T-Soil) and solar irradiance (Ra). The down arrow shows the date of fertilization (247 kg-N ha^{-1}, typical compound fertilizer used by local farmers, N: P_2O_5: K_2O=24: 12: 6), and the shallowed regions represent nighttime periods.

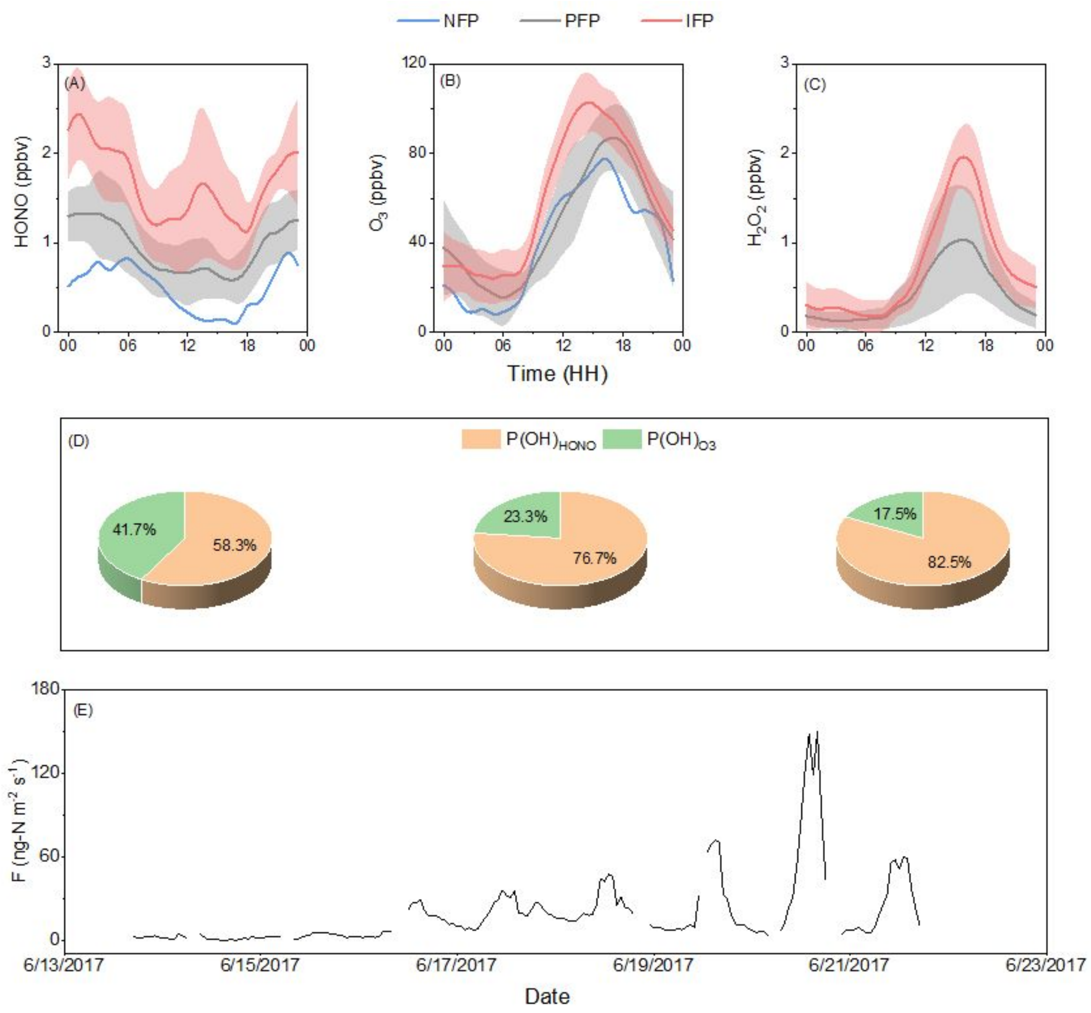

Figure S3: Average diurnal profiles of HONO (A), O_3 (B), and H_2O_2 (C) measured at the SRE-CAS site; (D): relative contributions of HONO and O_3 to primary OH production in the summer of 2017[6]. NFP: before fertilization period; PFP: pre-intensive fertilization; IFP: intensive fertilization period; (E): the measured HONO flux in 2017.

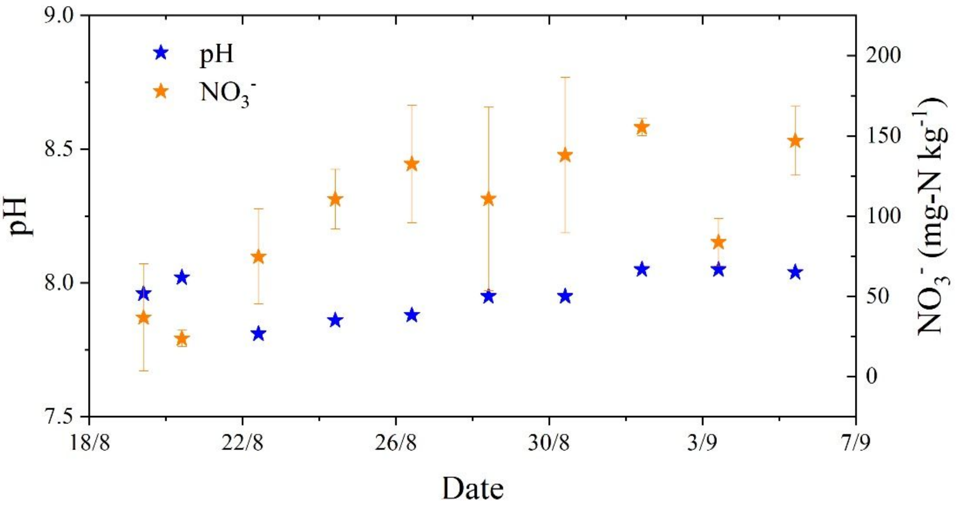

Figure S4: The measured soil pH and NO_3^- concentration during the campaign from 19 Aug. to 6 Sept. 2016. Error bars for the NO_3^- curve represent the standard deviation of three soil samples.

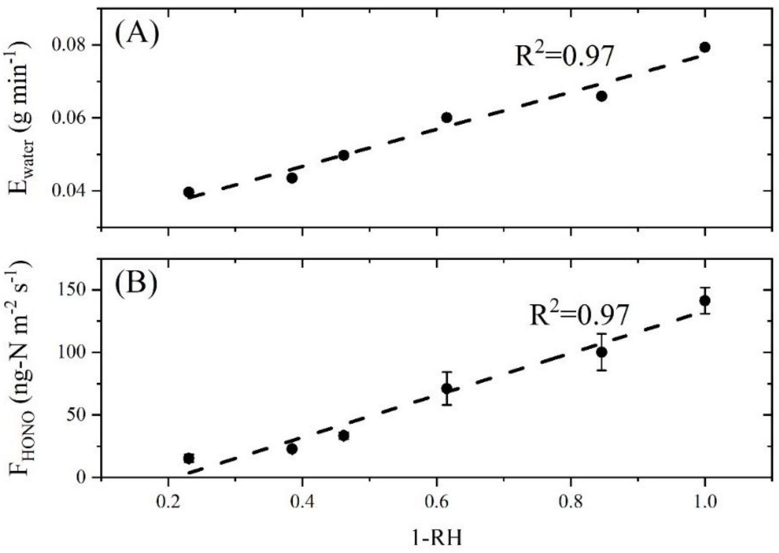

Figure S5: (A) The correlation between soil water loss rate (E_{water}) and the gap to saturated condition (1-RH) and (B) the correlation between the HONO flux (F_{HONO}) and 1-RH during the gradient RH experiment.

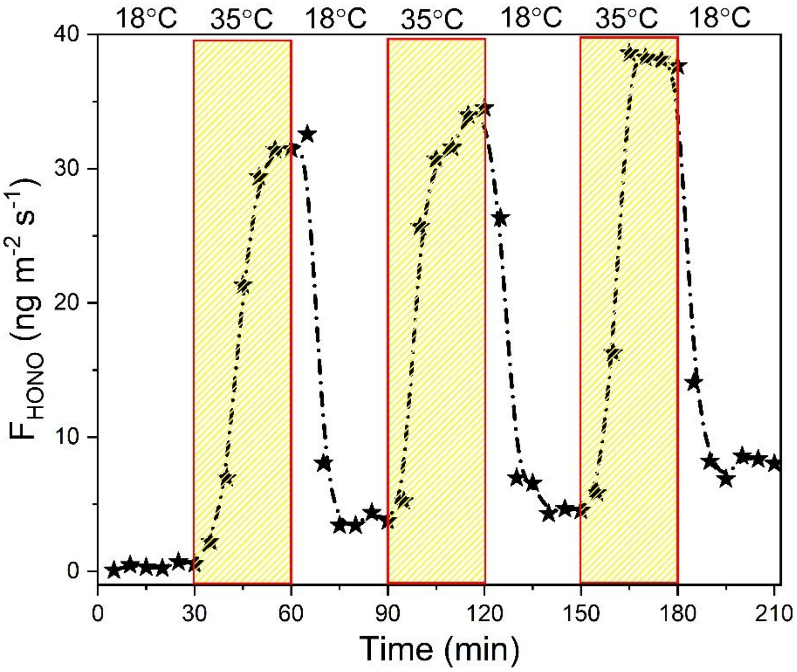

Figure S6: Results of the temperature dependence experiment.

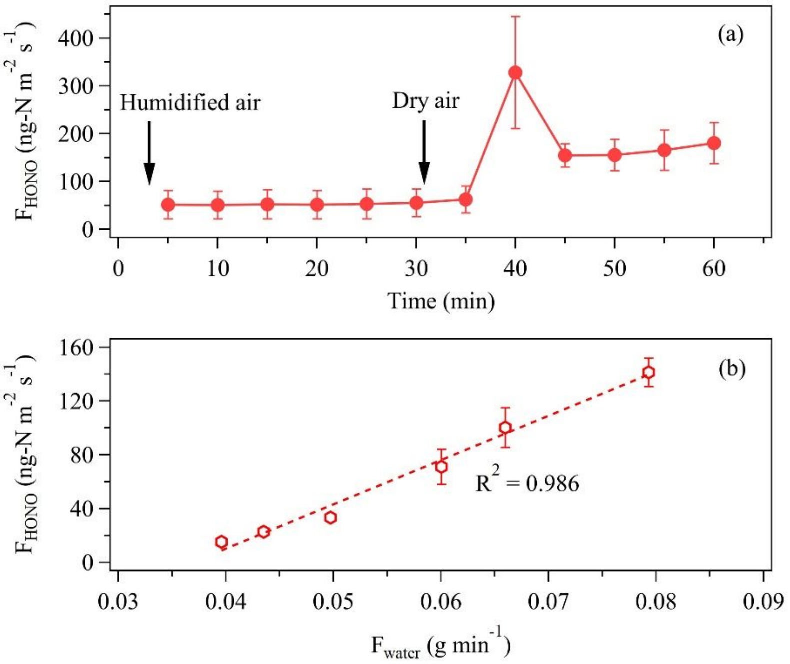

Figure S7: Influence of water evaporation on HONO emissions from NH_4Cl-fertilized soil sample. (a) The variation characteristic of F_{HONO} when switching flushing gas from the humidified air (RH: 48.8%) to dry air at a constant soil temperature of 35 °C. The error bars are the standard deviations for triplicate experiments; (b) The correlation between F_{HONO} and soil water loss rate (E_{water}) during the gradient RH experiment.

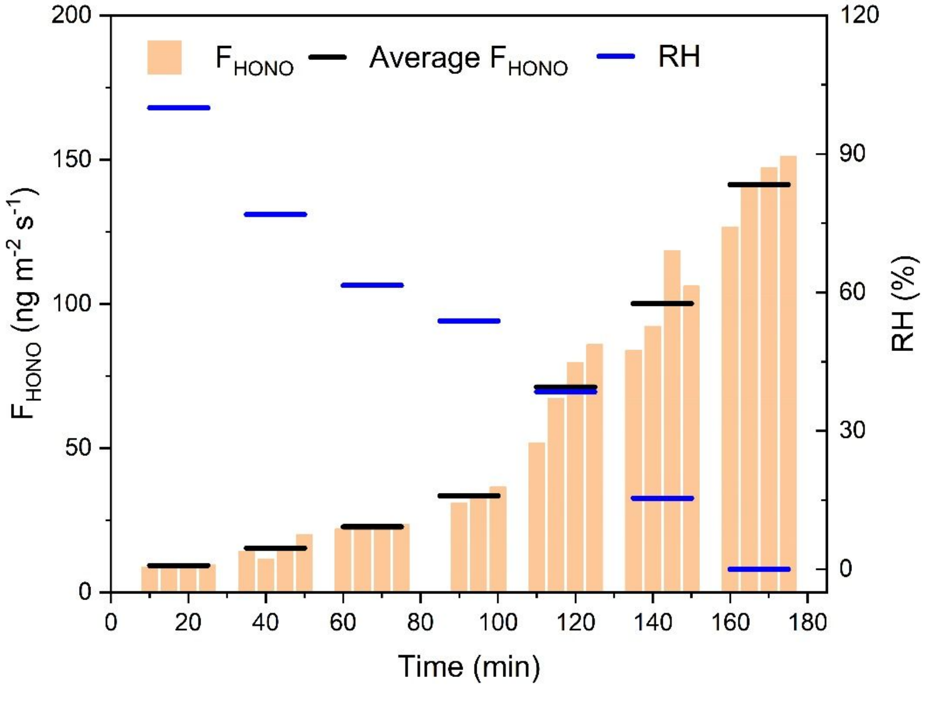

Figure S8: Time series of F_{HONO} measured during gradient RH experiments. The blue lines represent RH and the black lines represent the corresponding average F_{HONO}.

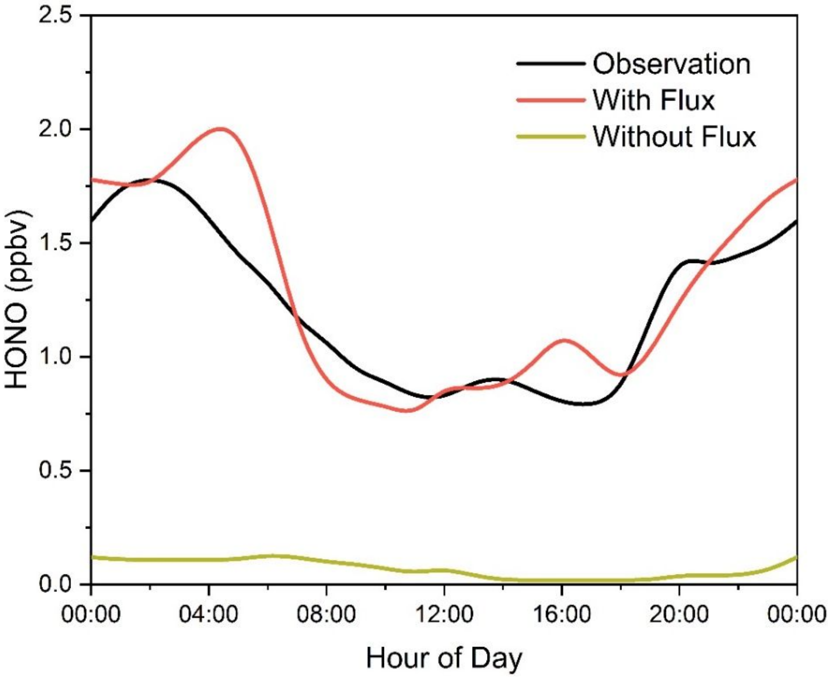

Figure S9: Results of model simulations with/without HONO flux in comparison with observations.

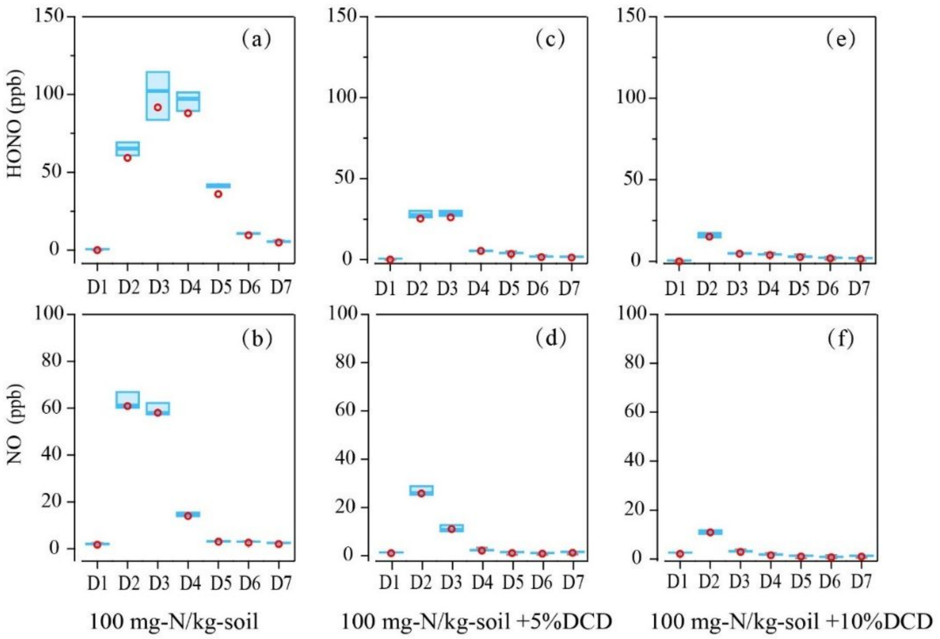

Figure S10: Impacts of a nitrification inhibitor (DCD) on soil HONO and NO emissions from the first day (D1) to the seventh day (D7). The top and the bottom of each box represent 25 and 75 percentiles of HONO or NO concentrations in the incubator with soil samples treated by NH_4Cl (a and b), NH_4Cl+5%DCD (c and d), or NH_4Cl+10%DCD (e and f). 5%DCD and 10%DCD denote that the application of DCD

represents 5% and 10% of the applied nitrogen by mass, respectively. The red dot and the blue line inside the box represent the median and average values, respectively. The x-axis represents the incubation day of the soil samples.

References

(1) Xue, C.; Ye, C.; Zhang, Y.; Ma, Z.; Liu, P.; Zhang, C.; Zhao, X.; Liu, J.; Mu, Y. Development and Application of a Twin Open-Top Chambers Method to Measure Soil HONO Emission in the North China Plain. *Sci. Total Environ.* **2019**, *659*, 621–631. https://doi.org/10.1016/j.scitotenv.2018.12.245.

(2) Song, Y.; Xue, C.; Zhang, Y.; Liu, P.; Bao, F.; Li, X.; Mu, Y. Measurement Report: Exchange Fluxes of HONO over Agricultural Fields in the North China Plain. *Atmos. Chem. Phys.* **2023**, *23* (24), 15733–15747. https://doi.org/10.5194/acp-23-15733-2023.

(3) Xue, C.; Ye, C.; Ma, Z.; Liu, P.; Zhang, Y.; Zhang, C.; Tang, K.; Zhang, W.; Zhao, X.; Wang, Y.; Song, M.; Liu, J.; Duan, J.; Qin, M.; Tong, S.; Ge, M.; Mu, Y. Development of Stripping Coil-Ion Chromatograph Method and Intercomparison with CEAS and LOPAP to Measure Atmospheric HONO. *Sci. Total Environ.* **2019**, *646*, 187–195. https://doi.org/10.1016/j.scitotenv.2018.07.244.

(4) Goliff, W. S.; Stockwell, W. R.; Lawson, C. V. The Regional Atmospheric Chemistry Mechanism, Version 2. *Atmos. Environ.* **2013**, *68* (x), 174–185. https://doi.org/10.1016/j.atmosenv.2012.11.038.

(5) Liu, Y.; Lu, K.; Li, X.; Dong, H.; Tan, Z.; Wang, H.; Zou, Q.; Wu, Y.; Zeng, L.; Hu, M.; Min, K.-E.; Kecorius, S.; Wiedensohler, A.; Zhang, Y. A Comprehensive Model Test of the HONO Sources Constrained to Field Measurements at Rural North China Plain. *Environ. Sci. Technol.* **2019**, *53* (7), 3517–3525. https://doi.org/10.1021/acs.est.8b06367.

(6) Xue, C.; Ye, C.; Zhang, C.; Catoire, V.; Liu, P.; Gu, R.; Zhang, J.; Ma, Z.; Zhao, X.; Zhang, W.; Ren, Y.; Krysztofiak, G.; Tong, S.; Xue, L.; An, J.; Ge, M.; Mellouki, A.; Mu, Y. Evidence for Strong HONO Emission from Fertilized Agricultural Fields and Its Remarkable Impact on Regional O3 Pollution in the Summer North China Plain. *ACS Earth Sp. Chem.* **2021**, *5* (2), 340–347. https://doi.org/10.1021/acsearthspacechem.0c00314.
